# BILIARY SLUDGE-ASSOCIATED ACUTE PANCREATITIS: CLINICAL SPECTRUM, DIAGNOSIS, AND MANAGEMENT

**DOI:** 10.1590/S0004-2803.24612025-155

**Published:** 2026-07-24

**Authors:** Mingqi SU, Yayun XIE, Ji CHEN

**Affiliations:** 1 Tongren Hospital, Shanghai Jiao Tong University School of Medicine, Department of General Surgery, Shanghai, China.

**Keywords:** Acute pancreatitis, biliary sludge, microlithiasis, endoscopic ultrasonography, cholecystectomy, ERCP, Pancreatite aguda, lama biliar, microlitíase, ultrassonografia endoscópica, colecistectomia, CPRE

## Abstract

**Background:**

Biliary disease is the leading identifiable cause of acute pancreatitis, yet many attacks remain “idiopathic” after routine tests. Wider use of endoscopic ultrasonography (EUS) has revealed biliary sludge and microlithiasis as frequent, previously occult causes of pancreatitis.

**Objective:**

To summarise current evidence on the pathophysiology, diagnosis and management of biliary sludge-associated acute pancreatitis (BSAP) and propose a simple clinical framework.

**Methods:**

Narrative review of experimental and clinical studies, practice guidelines and consensus statements identified through targeted searches of PubMed, Embase and the Cochrane Library, complemented by citation tracking of key articles.

**Results:**

Available data support a microcrystal-driven model in which lithogenic bile, impaired gallbladder emptying and mucin-rich aggregates promote sludge formation, while transient obstruction and bile acid-mediated epithelial injury in the distal bile duct-papillary outflow segment trigger pancreatitis. First-line ultrasonography and computed tomography have low sensitivity for sludge, whereas EUS is more sensitive than magnetic resonance cholangiopancreatography for detecting microlithiasis in suspected idiopathic acute pancreatitis. Grading diagnostic confidence (definite, probable or presumptive BSAP) and integrating disease severity can rationalise use of MRCP/EUS, selective ERCP, cholecystectomy and adjunctive medical or metabolic strategies.

**Conclusion:**

BSAP is a clinically relevant entity within the biliary pancreatitis spectrum. Applying a structured, confidence- and severity-based approach may help standardise investigations, optimise timing of ERCP and cholecystectomy and reduce preventable recurrences.

## HIGHLIGHTS

Biliary sludge and microlithiasis are frequent, under-recognized causes of “idiopathic” acute pancreatitis.

EUS is more sensitive than MRCP for detecting microlithiasis and can improve diagnostic confidence in suspected BSAP.

Integrating diagnostic confidence with Revised Atlanta severity helps tailor ERCP, cholecystectomy timing, and duct clearance strategies.

## RESUMO

**Contexto:**

A doença biliar é a principal causa identificável de pancreatite aguda, embora muitos episódios permaneçam “idiopáticos” após exames de rotina. O uso ampliado da ultrassonografia endoscópica (EUS) tem revelado a lama biliar e a microlitíase como causas frequentes, previamente ocultas, de pancreatite.

**Objetivo:**

Resumir as evidências atuais sobre a fisiopatologia, o diagnóstico e o manejo da pancreatite aguda associada à lama biliar (BSAP) e propor um modelo clínico simplificado.

**Métodos:**

Revisão narrativa de estudos experimentais e clínicos, diretrizes de prática clínica e consensos identificados por meio de buscas direcionadas nas bases PubMed, Embase e Cochrane Library, complementadas por rastreamento de citações de artigos-chave.

**Resultados:**

Os dados disponíveis sustentam um modelo mediado por microcristais, no qual a bile litogênica, o esvaziamento vesicular prejudicado e agregados ricos em mucina promovem a formação de lama biliar, enquanto a obstrução transitória e a lesão epitelial mediada por ácidos biliares no segmento distal do ducto biliar/fluxo papilar desencadeiam pancreatite. A ultrassonografia e a tomografia computadorizada de primeira linha apresentam baixa sensibilidade na detecção de lama biliar, ao passo que a EUS é mais sensível do que a colangiopancreatografia por ressonância magnética (CPRM) para detectar microlitíase em casos suspeitos de pancreatite aguda idiopática. A classificação do grau de confiança diagnóstica (definida, provável ou presumida BSAP) e a integração com a gravidade da doença podem racionalizar o uso de CPRM/EUS, CPRE seletiva, colecistectomia e estratégias médicas ou metabólicas adjuvantes.

**Conclusão:**

A BSAP é uma entidade clinicamente relevante no espectro da pancreatite biliar. A aplicação de uma abordagem estruturada, baseada na confiança diagnóstica e na gravidade, pode ajudar a padronizar a investigação, otimizar o momento da CPRE e da colecistectomia e reduzir recorrências evitáveis.

## INTRODUCTION

Acute pancreatitis is one of the most common causes of gastrointestinal admission worldwide^1^. Biliary disease accounts for approximately 40-70% of episodes and remains the leading identified aetiology^2^. Even after a standard work-up with serum biochemistry, transabdominal ultrasonography and computed tomography, 16-27% of attacks are still classified as idiopathic acute pancreatitis^2^ ^,^ ^3^ ^,^ ^4^.

With increasing use of endoscopic ultrasonography (EUS), many of these “idiopathic” cases are now attributed to biliary sludge or microlithiasis - microcrystalline material within the gallbladder or bile duct that cannot be reliably detected by conventional ultrasonography or computed tomography^5^ ^,^ ^6^ ^,^ ^7^. Consensus statements emphasise that sludge and microlithiasis form a microcrystal continuum, share similar risk factors and mechanisms, and may independently trigger hepatopancreatobiliary disease^5^ ^,^ ^8^.

In this context, it is useful to describe biliary sludge-associated acute pancreatitis (BSAP) as acute pancreatitis of presumed biliary origin in the absence of duct-occluding macrolithiasis, where the most plausible mechanism is transient obstruction and biliary-pancreatic reflux induced by sludge or microlithiasis. This concept encompasses patients previously labelled as idiopathic acute pancreatitis and patients with a “biliary phenotype” but stone-negative imaging. Emerging data suggest that BSAP behaves differently from classic gallstone pancreatitis, with a tendency towards milder disease and fewer long-term pancreatobiliary complications^9^ ^,^ ^10^.

This narrative review summarises current evidence on the pathophysiology, diagnostic evaluation and management of BSAP. Building on recent consensus statements and pancreatitis guidelines, it proposes a simple, confidence- and severity-based framework intended for everyday practice rather than as a formal guideline^3^ ^,^ ^4^ ^,^ ^5^.

## METHODS

This article is a narrative, non-systematic review. We searched PubMed, Embase and the Cochrane Library from inception to 30 September 2025, using controlled vocabulary and free-text terms related to biliary sludge, microlithiasis, biliary pancreatitis, idiopathic acute pancreatitis, endoscopic ultrasonography, MRCP, ERCP, cholecystectomy and ursodeoxycholic acid (example PubMed search: (“biliary sludge” OR “microlithiasis”) AND (“pancreatitis” OR “idiopathic acute pancreatitis”)). Only English-language human studies were considered. We included experimental and clinical studies, practice guidelines, consensus statements and high-quality narrative reviews reporting data on pathophysiology, diagnostic performance of imaging, or outcomes of endoscopic, surgical or pharmacologic management; case reports and very small series were used only illustratively. Titles and abstracts were screened by one author, with a second author consulted for uncertain cases; screening was not performed in duplicate. Full texts were reviewed when needed, and reference lists of key articles were hand-searched to identify additional studies. Randomised trials, meta-analyses and international guidelines were prioritised when formulating recommendations. No formal risk-of-bias assessment or quantitative synthesis was performed, consistent with the narrative review scope. The search strategies for all databases are provided in APPENDIX A.

**APPENDIX A:**
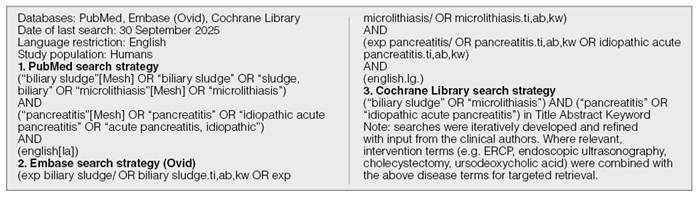
Search strategy.

### Pathophysiology of biliary sludge-associated pancreatitis

### Formation of sludge and microlithiasis

Biliary sludge is now defined, in accordance with the recent international consensus definition on sludge and microlithiasis as a possible cause of pancreatitis, as discrete hyperechoic material within the gallbladder or bile ducts that sediments in the most dependent part without generating an acoustic shadow^5^. Biliary microlithiasis is defined as calculi in the gallbladder or bile ducts measuring ≤5 mm in diameter that display acoustic shadowing on ultrasonography^5^. In the systematic review underpinning this consensus, approximately 13% of original studies and 19% of review articles used the terms “sludge” and “microlithiasis” interchangeably, and there was wide variation in size cut-offs and sonographic criteria, which limits comparability of reported prevalence and pancreatitis risk between cohorts^5^.

On a pathophysiological level, sludge and microlithiasis form when bile becomes supersaturated with cholesterol or calcium bilirubinate and gallbladder emptying is impaired, favouring nucleation of microcrystals, aggregation with mucins and retention within more viscous bile^5^ ^,^ ^11^. Established risk factors include pregnancy, rapid weight loss, prolonged fasting or total parenteral nutrition and selected medications such as ceftriaxone and octreotide^5^ ^,^ ^8^ ^,^ ^12^. Metabolic dysfunction-associated steatotic liver disease, obesity and insulin resistance further alter bile composition, reduce gallbladder motility and promote local inflammation, thereby increasing the risk of sludge and gallstone formation^8^ ^,^ ^13^ ^,^ ^14^. Alterations in the gut microbiome may also influence bile acid metabolism and enterohepatic circulation^15^.

Given their shared pathogenesis and overlapping clinical implications, most authors now regard sludge and microlithiasis as part of a continuous microcrystal spectrum. In this review, the term “biliary sludge” is used pragmatically to encompass both layered sludge and microlithiasis unless a distinction is specifically relevant.

### From microcrystals to pancreatic injury

Experimental and clinical data suggest that BSAP arises from a combination of intermittent obstruction and bile-acid-mediated epithelial injury^16^ ^,^ ^17^. Three inter-related processes appear particularly important.

First, migration of sludge or microcrystals into the distal common bile duct and papilla can produce short-lived obstruction, ductal hypertension and biliary-pancreatic reflux. This is especially relevant when soft sludge and viscous bile accumulate in the distal bile duct-papillary segment, narrowing the outflow tract without a fixed stone^5^ ^,^ ^6^. Attacks may resolve spontaneously as the material passes or redistributes.

Second, bile acids, lysolecithin and microcrystals directly injure pancreatic duct and acinar cells, disturbing calcium homeostasis and activating inflammatory pathways such as calcineurin and innate immune receptors^16^ ^,^ ^17^ ^,^ ^18^. Repeated low-grade exposure can “prime” the gland, favouring recurrent rather than fulminant attacks^9^ ^,^ ^16^. Papillary oedema and sphincter dysfunction may further perpetuate impaired drainage.

Third, mucin overproduction and crystal deposition increase bile viscosity and may activate NLRP3 inflammasomes, contributing to a self-perpetuating cycle of sludge stabilisation and local inflammation^11^ ^,^ ^19^. These mechanisms help explain common clinical observations: brief cholestatic enzyme flares, negative initial imaging and a tendency to recurrent episodes when the biliary source remains untreated^9^ ^,^ ^10^.

### Clinical presentation and diagnostic evaluation

### Clinical and laboratory features

The clinical picture of BSAP is indistinguishable from classical gallstone pancreatitis in most patients. Typical features include acute epigastric or right upper quadrant pain radiating to the back, often accompanied by nausea or vomiting^2^ ^,^ ^3^.

Biochemically, a transient rise in aminotransferases and cholestatic enzymes (alkaline phosphatase and γ-glutamyl transferase) suggests short-lived biliary obstruction rather than sustained impaction^2^. Amylase and lipase are elevated but non-specific, and systemic inflammatory markers reflect disease severity rather than aetiology^3^. In practice, the combination of a biliary-type pain pattern, cholestatic enzyme flare and stone-negative first-line imaging should prompt evaluation for occult sludge or microlithiasis.

### Limitations of first-line imaging

Transabdominal ultrasonography is the first-line imaging test in suspected biliary or pancreatic disease because it is inexpensive, non-invasive and widely available^2^. Under optimal conditions it reliably detects gallbladder stones and secondary signs of biliary obstruction. However, in emergency settings, patient-related factors such as a non-fasted state, bowel gas, pain-limited inspiration and obesity often reduce acoustic windows and diagnostic confidence^2^.

Non-contrast computed tomography is frequently performed to evaluate severity and alternative diagnoses. While useful for staging peripancreatic collections and necrosis, CT is poorly sensitive for sludge and small ductal stones, which are often iso-dense to bile. As a result, conventional ultrasonography and CT frequently miss sludge and microlithiasis, justifying a staged work-up when the clinical scenario suggests BSAP^5^ ^,^ ^20^.

### MRCP and EUS: complementary roles

Magnetic resonance cholangiopancreatography (MRCP) provides a non-invasive overview of the biliary tree and pancreatic duct. Recent advances-including breath-hold compressed-sensing acquisitions, high-resolution 3D T2-weighted imaging at 3 T and secretin-enhanced protocols-have improved duct conspicuity and depiction of subtle filling defects^21^ ^,^ ^22^.

Nevertheless, endoscopic ultrasonography (EUS) consistently outperforms MRCP for detecting microlithiasis, sludge and stones smaller than 5-6 mm, particularly in patients with idiopathic acute pancreatitis. In a systematic review and meta-analysis of idiopathic acute pancreatitis, EUS identified an underlying aetiology in approximately 64% of patients, compared to about 34% with MRCP, demonstrating a superior diagnostic yield while maintaining comparable specificity^20^. Single-centre comparative data, including secretin-enhanced MRCP, similarly support the superior diagnostic yield of EUS in this setting^23^. EUS also enables targeted inspection of the papilla and periampullary region and can directly guide subsequent ERCP when indicated^7^ ^,^ ^24^.

In practice, MRCP is generally preferred as the first non-invasive step when available, whereas EUS-the most sensitive test for occult biliary aetiology-is recommended after non-diagnostic MRCP or when clinical suspicion remains high despite negative imaging^7^ ^,^ ^20^ ^,^ ^23^.

### A simple diagnostic pathway

For routine care, it is helpful to combine clinical probability, local resources and diagnostic confidence into a simple pathway. This review focuses on patients who meet standard criteria for acute pancreatitis, have clinical or biochemical features suggestive of biliary origin, but show no gallstones or choledocholithiasis on initial ultrasonography and CT^2^ ^-^ ^4^.

In stable patients with high clinical suspicion and access to advanced imaging, an MRCP-first, EUS-second strategy is a pragmatic option. MRCP provides an anatomical overview, while EUS is reserved for negative or equivocal MRCP results or when high-resolution assessment is required^20^ ^,^ ^23^. However, no randomised trials have directly compared MRCP-first with EUS-first strategies in terms of clinical outcomes or cost-effectiveness in suspected BSAP or idiopathic acute pancreatitis, and current practice remains heterogeneous, being largely shaped by local expertise, resource availability and extrapolation from observational cohorts, meta-analyses and narrative reviews that underline the need for prospective comparative studies^4^ ^,^ ^7^ ^,^ ^20^ ^,^ ^24^.

Where EUS is unavailable, MRCP remains the main tool; persistent uncertainty may justify repeat imaging or referral. In resource-limited settings without MRCP or EUS, diagnostic ERCP may be considered in severe presentations, whereas conservative treatment with close monitoring is appropriate in stable patients^24^.

For decision-making, clinicians may classify BSAP according to diagnostic confidence:

Definite BSAP: direct visualisation of sludge or microlithiasis on EUS or ERCP.

Probable BSAP: compatible clinical picture plus suggestive MRCP findings (for example, ductal debris or dilation) but without direct endoscopic confirmation.

Presumptive BSAP: strong clinical and biochemical suspicion, but all available imaging is non-diagnostic.

In addition, disease severity should be graded using the Revised Atlanta classification (mild, moderate and severe), independent of aetiology^3^. Together, diagnostic confidence (definite, probable or presumptive BSAP) and severity (mild, moderate or severe) form the basis for guiding management intensity and timing.

TABLE 1 outlines a simplified, scenario-based diagnostic pathway that can be adapted to local resources. 

**TABLE 1:** Scenario-based diagnostic pathway for suspected biliary sludge-associated pancreatitis (BSAP).

Clinical scenario	Preferred diagnostic sequence	Primary objective
Scenario 1 - Stable patient, high clinical suspicion (most common)	1) MRCP. 2) EUS if MRCP is negative or indeterminate.	To establish a biliary microcrystal aetiology and plan definitive therapy.
Scenario 2 - EUS unavailable or declined (less common)	MRCP as the primary imaging tool; consider repeat MRCP or referral for EUS if diagnostic uncertainty persists.	To identify indirect signs of microlithiasis and stratify the risk of recurrence.
Scenario 3 - Resource-limited setting (less common)	If expertise is available and symptoms are severe or deteriorating: diagnostic ERCP (with potential for immediate therapy). Otherwise: conservative management with a predefined plan for transfer if the clinical condition worsens.	To establish a diagnosis with the option of immediate treatment, or to stabilise the patient and safely escalate care.
Scenario 4 - Acute cholangitis or progressive obstruction (time-critical)	Emergency or urgent ERCP for biliary decompression.	To control sepsis, relieve biliary obstruction and confirm the underlying aetiology.

US: ultrasonography. CT: computed tomography. MRCP: magnetic resonance cholangiopancreatography. EUS: endoscopic ultrasonography. ERCP: endoscopic retrograde cholangiopancreatography. BSAP: biliary sludge-associated acute pancreatitis.

For every patient, clinicians should record diagnostic confidence, highest level of diagnostic evidence (EUS, ERCP, MRCP or clinical only) and disease severity. This simple documentation creates a reproducible link between diagnosis and management planning and may support future research on BSAP.

### Management strategies

### General principles and severity-stratified management

Management of BSAP follows the core principles of acute biliary pancreatitis but must account for its intermittent, microcrystal-driven nature^3^ ^,^ ^9^. Although BSAP often presents as mild-to-moderate disease, comparative data suggest similar severity between sludge/microlithiasis-induced and classic gallstone pancreatitis, while subsequent pancreatobiliary complications appear less frequent in sludge/microlithiasis cohorts^9^ ^,^ ^10^.

Initial treatment - fluid resuscitation, adequate analgesia, early enteral nutrition and organ support when required - mirrors general acute pancreatitis guidelines^3^. Indications for ERCP and subsequent invasive management are detailed in Sections 5.2-5.3.

Source control should be planned according to severity and diagnostic confidence. In patients with definite or probable BSAP and a gallbladder in situ, definitive treatment usually involves laparoscopic cholecystectomy (LC) with or without common bile duct evaluation^3^ ^,^ ^25^ ^,^ ^26^. Presumptive BSAP without gallbladder pathology may be managed conservatively, with reassessment using EUS or MRCP in case of recurrence^4^.

### Endoscopic management

ERCP has a selective, rather than routine, role in BSAP (Table 2)^3^ ^,^ ^24^ ^,^ ^25^. Urgent ERCP (within 24 hours) is indicated for acute cholangitis or clear evidence of persistent biliary obstruction. In stable patients with suspected BSAP, ductal imaging should preferentially be achieved by MRCP and/or EUS, reserving ERCP for cases where duct clearance is expected to change management^7^ ^,^ ^20^ ^,^ ^24^ ^,^ ^25^.

**TABLE 2:** Practical recommendations with evidence certainty and uncertainty.

Domain	Practical recommendation (key references)	Evidence certainty*	Key uncertainty / notes (brief)
**Diagnostic confirmation (MRCP/EUS)**	After negative initial US/CT in biliary-phenotype acute pancreatitis, evaluate for occult sludge/microlithiasis with MRCP and/or EUS; a pragmatic MRCP-first then EUS approach is reasonable where resources allow^4^ ^,^ ^7^ ^,^ ^20^ ^,^ ^23^ ^.^	Moderate	No RCTs comparing sequential strategies on clinical outcomes; yield depends on timing, definitions, and operator expertise.
**Endoscopic ductal intervention**	Reserve urgent ERCP for acute cholangitis or clear evidence of persistent biliary obstruction; avoid routine ERCP in uncomplicated BSAP^3^ ^,^ ^24^ ^,^ ^25^.	High (for cholangitis/obstruction); Moderate (against routine ERCP)	Thresholds for “persistent obstruction” vary across centres; benefit-risk depends on local expertise.
	For sludge-predominant ductal disease, plan ERCP with an outflow-targeted, sphincter-preserving strategy (e.g., limited sphincterotomy ± low-pressure balloon dilation; gentle clearance)^24^ ^,^ ^25^ ^,^ ^28^.	Low-Moderate	Technique- and operator-dependent; comparative outcomes data remain limited.
	Short-term biliary drainage: consider ENBD for unstable patients (allows decompression/irrigation) versus internal plastic stent for stable patients (convenience; occlusion/migration risk)^29^.	Moderate	Choice is context-dependent (tolerance, infection control needs, and follow-up logistics).
	Use guideline-based PEP prophylaxis per ESGE/ASGE: rectal NSAIDs for most patients, with prophylactic pancreatic stenting in selected high-risk cases^30^ ^,^ ^31^.	High	Risk stratification varies; implementation depends on local protocols and operator experience.
**Definitive source control (LC timing)**	Mild BSAP: perform same-admission LC (typically within 2-7 days once clinically stable); supported indirectly by the PONCHO RCT in mild gallstone pancreatitis^32^ and observational data after ERCP^33^.	High (indirect RCT evidence)	BSAP-specific RCTs are lacking; applicability inferred from mild gallstone pancreatitis.
	Moderate disease: consider early elective LC (~1-3 weeks) after inflammation subsides^34^. Severe/necrotising disease: delay LC (~6-8 weeks) to allow collections/necrosis to stabilise^35^.	Moderate	Timing should be individualised (collections maturity, comorbidity, operative risk).
**Adjunct / supportive care**	Consider UDCA only as adjunct/bridge in patients unfit for or declining invasive source control^26^ ^,^ ^38^ ^,^ ^39^; encourage early feeding and longer-term metabolic/lifestyle optimisation^41^ ^,^ ^42^ ^,^ ^8^ ^,^ ^12^ ^-^ ^14^.	Low-Moderate	BSAP-specific data are limited; benefits and optimal duration are uncertain.

* Evidence certainty (simplified; not a formal GRADE assessment)**:** High = RCTs/high-quality meta-analyses (or strong guideline-based evidence); Moderate = observational/indirect evidence with limitations; Low = limited observational evidence or expert opinion.

ASGE: American Society for Gastrointestinal Endoscopy. ESGE: European Society of Gastrointestinal Endoscopy. PEP: post-ERCP pancreatitis. NSAIDs: non-steroidal anti-inflammatory drugs. ENBD: endoscopic nasobiliary drainage. BSAP: biliary sludge-microlithiasis-associated pancreatitis. US: ultrasonography. CT: computed tomography. MRCP: magnetic resonance cholangiopancreatography. EUS: endoscopic ultrasonography. ERCP: endoscopic retrograde cholangiopancreatography. LC: laparoscopic cholecystectomy. UDCA: ursodeoxycholic acid. RCT: randomised controlled trial. PONCHO: trial of same-admission vs interval cholecystectomy in mild gallstone pancreatitis.

Technical refinements aim to minimise procedure-related injury.Wire-guided (rather than contrast-guided) cannulation reduces the risk of post-ERCP pancreatitis^27^. Limited sphincterotomy combined with low-pressure balloon dilation facilitates clearance of soft sludge while preserving papillary function^25^. Gentle irrigation and staged clearance are preferred over aggressive single-session attempts^25^ ^,^ ^28^.

In sludge-predominant BSAP, ERCP should be planned with a specific focus on the distal common bile duct and papillary outflow segment. Techniques that combine small-incision sphincterotomy, controlled balloon dilation and repeated balloon sweeping or aspiration can clear obstructing sludge, viscous bile and microcrystals in this outflow tract while maintaining as much sphincter function as possible^24^ ^,^ ^25^. This “outflow-targeted, sphincter-preserving” strategy is conceptually distinct from classical large-incision sphincterotomy used for firm stones and is particularly suited to patients whose imaging suggests poorly defined debris rather than discrete calculi.

Short-term drainage strategies should be individualised. Nasobiliary drainage allows continuous decompression and irrigation in unstable patients, whereas internal plastic stents offer convenience in stable patients but carry risks of occlusion and migration^29^. Standard prophylaxis against post-ERCP pancreatitis - including rectal non-steroidal anti-inflammatory drugs and selective pancreatic stenting in high-risk cases - is recommended according to current ESGE and ASGE guidelines^30^ ^,^ ^31^.

Emerging modalities, such as digital single-operator cholangioscopy and biodegradable stents, may be useful in complex sludge-predominant disease, but evidence remains limited and largely extrapolated from stone and stricture cohorts^28^.

### Surgical management and timing of cholecystectomy

LC is the cornerstone of definitive source control, eliminating the primary reservoir of sludge and microlithiasis^26^. Timing is dictated by disease severity and local expertise.

In mild BSAP, same-admission LC (within 2-7 days after stabilisation) reduces recurrent biliary events and is consistent with randomised data in mild gallstone pancreatitis. In the PONCHO multicentre randomised controlled trial (Lancet 2015), same-admission cholecystectomy reduced readmission for recurrent gallstone-related complications or death within 6 months compared with interval surgery (5% vs 17%; risk ratio 0.28, 95%CI 0.12-0.66; *P*=0.002), with a very low risk of major cholecystectomy-related complications^3^ ^,^ ^32^. In patients who undergo ERCP for cholangitis or persistent obstruction, performing LC during the same admission further reduces recurrent events and unplanned readmissions^33^.

In moderate disease, early elective LC (approximately 1-3 weeks after the acute episode) is feasible in improving patients when local inflammation is controlled and operative risk is acceptable^34^. In severe disease, a delayed strategy, typically 6-8 weeks after onset, is preferred to allow collections to mature and necrosis to stabilise^35^.

Where ductal sludge or stones are suspected, centres with appropriate expertise may offer one-stage LC plus laparoscopic common bile duct exploration (LCBDE). Meta-analyses show that LCBDE achieves duct clearance and long-term outcomes comparable to ERCP plus LC in mixed stone cohorts, although it is more operator-dependent^36^ ^,^ ^37^. ERCP-first strategies remain appropriate when LCBDE expertise is limited; early conversion to staged ERCP should be favoured over escalating surgical risk^25^ ^,^ ^36^.

In sludge-predominant disease, the balance between LCBDE and ERCP deserves particular consideration. Transcystic or transcholedochal LCBDE is well suited for discrete, impacted stones, but soft sludge, viscous bile and microcrystals located in the distal common bile duct or at the papilla can be difficult to clear completely with baskets or balloons introduced from above. Residual debris at the terminal duct segment may continue to impair outflow even after an apparently successful exploration. By contrast, ERCP using small-incision sphincterotomy and/or low-pressure balloon dilation allows direct treatment of the outflow segment - the terminal common bile duct and papilla - with sweeping, aspiration and irrigation of obstructing sludge and other outflow-limiting factors^25^. When limited-incision or sphincter-preserving techniques are used, papillary barrier function can be largely maintained while still removing the sludge burden. In practice, this makes ERCP particularly attractive in patients with mildly dilated ducts, poorly defined stones and a functional or inflammatory papillary component coexisting with biliary sludge.

In centres with both strong ERCP and LCBDE expertise, a flexible, patient-tailored strategy - using LCBDE for discrete stones and an ERCP-based, outflow-targeted approach for sludge-predominant BSAP - may provide the best balance between efficacy, papillary protection and resource use.

### Pharmacologic and supportive measures

Pharmacologic therapy is adjunctive in BSAP and cannot replace endoscopic or surgical source control when indicated. Ursodeoxycholic acid (UDCA) reduces bile lithogenicity and may dissolve small cholesterol stones or sludge over months^26^ ^,^ ^38^. However, direct evidence in BSAP is scarce; to our knowledge, no trials have evaluated BSAP-specific recurrence outcomes, and much of the clinical rationale is extrapolated from symptomatic gallstone disease and cholesterol microlithiasis, with modest and heterogeneous effects^39^.

UDCA can be considered in patients unfit for, or declining, invasive procedures, and as short-term chemoprevention after ERCP or LC in selected high-risk patients (for example, for 3-6 months)^26^ ^,^ ^39^. Typical dosing is 10-15 mg/kg/day with periodic monitoring. Responses are variable, and relapse after discontinuation is common^38^ ^,^ ^39^. Future agents targeting bile acid signalling pathways, such as farnesoid X receptor-FGF19 agonists or modulators of the gut microbiome, may further modify bile composition and inflammation, but current evidence remains preliminary^40^.

Nutritional and metabolic optimisation are important throughout the disease course. Early oral or enteral feeding according to enhanced recovery principles is safe in most patients after ERCP or LC and may shorten hospital stay^41^ ^,^ ^42^. Over the longer term, a Mediterranean-style, anti-inflammatory diet, weight optimisation, avoidance of very rapid weight loss and regular physical activity may help reduce sludge and stone formation, although BSAP-specific data are limited^8^ ^,^ ^12^ ^-^ ^14^.

### Practical uptake: recommendations and simplified pathway

**FIGURE 1:**
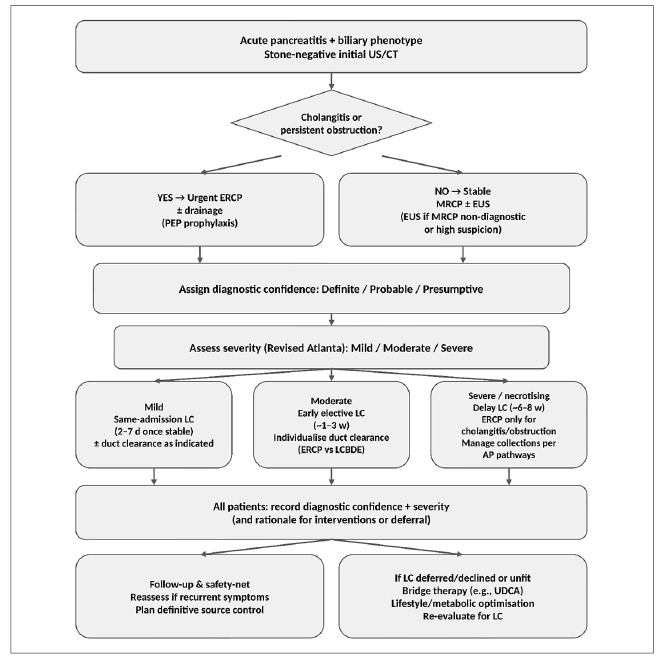
Simplified, printable clinical decision flowchart for suspected biliary sludge-associated acute pancreatitis.

To support clinical uptake, Table 2 summarises key practical recommendations for BSAP, together with evidence certainty and principal uncertainties. Figure 1 provides a simplified decision pathway that integrates diagnostic confidence and disease severity to operationalise these recommendations and guide investigation and timing of interventions.

AP: acute pancreatitis. ERCP: endoscopic retrograde cholangiopancreatography. EUS: endoscopic ultrasonography. LCBDE: laparoscopic common bile duct exploration. LC: laparoscopic cholecystectomy. MRCP: magnetic resonance cholangiopancreatography.

The flowchart summarises key decision steps for suspected biliary sludge-associated acute pancreatitis, including a bridge/alternative pathway when cholecystectomy is deferred, declined, or not feasible.

## CONCLUSION

Biliary sludge and microlithiasis are increasingly recognised as clinically important causes of acute pancreatitis. Many patients previously labelled as idiopathic in fact have biliary sludge-associated acute pancreatitis (BSAP), a microcrystal-driven subtype characterised by transient obstruction and bile-acid-mediated injury rather than fixed ductal blockage^5^ ^,^ ^6^ ^,^ ^9^.

This narrative review proposes a pragmatic framework for BSAP built on two pillars. The first is diagnostic confidence-definite, probable, or presumptive BSAP-derived from the best available combination of clinical, biochemical, and imaging data. The second is disease severity, assessed with the Revised Atlanta classification, which guides the intensity and timing of interventions^3^ ^,^ ^4^. Together, these domains can support rational use of MRCP and EUS, selective ERCP, timely cholecystectomy, and tailored use of laparoscopic common bile duct exploration (LCBDE) and pharmacologic adjuncts^3^ ^,^ ^5^ ^,^ ^9^ ^,^ ^10^ ^,^ ^24^ ^-^ ^26^.

In sludge-predominant BSAP, particular emphasis should be placed on targeted treatment of the distal bile duct-papillary outflow segment and on techniques that preserve papillary barrier function while effectively clearing sludge and microcrystals^24^ ^,^ ^25^.

Key research priorities must now be explicitly addressed to translate this framework into evidence-based practice, including:

Pragmatic head-to-head trials or prospective comparative studies comparing MRCP-first versus EUS-first strategies (including sequential algorithms) after negative initial ultrasonography/CT in suspected BSAP, as direct comparative evidence (including randomised data on clinical outcomes and cost-effectiveness) remains lacking. Key outcomes should include recurrent biliary events, need for therapeutic ERCP, time to definitive source control, adverse events, resource utilisation, and cost-effectiveness.

Longitudinal cohort studies to clarify the long-term impact of detecting biliary sludge or microlithiasis and whether detection-driven escalation (endoscopic intervention, cholecystectomy, adjunct medical therapy) reduces recurrence and other pancreatobiliary complications.

Health economic and access analyses, particularly for EUS in resource-limited settings, to inform scalable, equitable diagnostic pathways and sustainable service planning/referral models.

By framing BSAP as a distinct, mechanism-informed entity within the biliary pancreatitis spectrum, clinicians can move beyond a diagnosis of exclusion toward more structured, evidence-informed, and resource-sensitive care.

## Data Availability

not applicable.
